# Variational ecology and the physics of sentient systems

**DOI:** 10.1016/j.plrev.2018.12.002

**Published:** 2019-12

**Authors:** Maxwell J.D. Ramstead, Axel Constant, Paul B. Badcock, Karl J. Friston

**Affiliations:** a Division of Social and Transcultural Psychiatry, Department of Psychiatry, McGill University, Montreal, QC, H3A 1A1, Canada; b Department of Philosophy, McGill University, Montreal, QC, H3A 2T7, Canada; c Wellcome Trust Centre for Neuroimaging, University College London, London, WC1N 3BG, UK; d Amsterdam Brain and Cognition Center, The University of Amsterdam, Amsterdam, 1098 XH, the Netherlands; e Melbourne School of Psychological Sciences, The University of Melbourne, Melbourne, 3010, Australia; f Centre for Youth Mental Health, The University of Melbourne, Melbourne, 3052, Australia; g Orygen, the National Centre of Excellence in Youth Mental Health, Melbourne, 3052, Australia

**Keywords:** Physics of the mind, Free energy principle, Evolutionary systems theory, Variational neuroethology, Variational ecology, Niche construction

## Abstract

This paper addresses the challenges faced by multiscale formulations of the variational (free energy) approach to dynamics that obtain for large-scale ensembles. We review a framework for modelling complex adaptive control systems for multiscale free energy bounding organism–niche dynamics, thereby integrating the modelling strategies and heuristics of variational neuroethology with a broader perspective on the ecological nestedness of biotic systems. We extend the multiscale variational formulation beyond the action–perception loops of individual organisms by appealing to the variational approach to niche construction to explain the dynamics of coupled systems constituted by organisms and their ecological niche. We suggest that the statistical robustness of living systems is inherited, in part, from their eco-niches, as niches help coordinate dynamical patterns across larger spatiotemporal scales. We call this approach variational ecology. We argue that, when applied to cultural animals such as humans, variational ecology enables us to formulate not just a physics of individual minds, but also a physics of interacting minds across spatial and temporal scales – a physics of sentient systems that range from cells to societies.

## Highlights

• We extend the multiscale variational free energy approach to large-scale ensembles.
• We integrate multiscale modelling with a broad perspective on ecological nestedness.
• We argue that the statistical robustness of living systems is ecologically inherited.
• We propose variational ecology as a physics of sentient systems.

## 0 Introduction

Recent decades have witnessed the emergence of a new project for a *physics of the mind*. This effort has leveraged the constructs, principles, and methods of theoretical and experimental physics to investigate and understand what we call sentience and the ‘mind’. At the turn of the century, a new evolutionary systems theory of complex adaptive systems was proposed, made possible by the advent of computational modelling, variational methods of inference, and machine learning: the (variational) free energy principle (FEP) [1], [2], [3].

According to the FEP, the physics of sentient systems follows from the statistical mechanics of life. This variational formulation stems from the observation that living systems, over time and on average, tend to revisit the same set of *attracting* or *characteristic states*. These can be cast as the characteristic *phenotypic states* (and *traits*) of the organism. The FEP explains the dynamics of *any random dynamical system* that appears to resist decay through *adaptive action* [3], [4]. Under the FEP, organisms engage the environment in a self-fulfilling prophecy of sorts; ‘surfing’ up probability gradients towards their most probable phenotypic states [5]. The FEP was originally proposed in computational neuroscience to explain neural dynamics [6], [7], where it coheres broadly with predictive coding approaches [8], [9], [10], [11], and is widely recognised as a unifying theory of the function, structure, and dynamics of the brain [5], [12], [13], [14]. It has since been extended to explain the dynamics of biological systems within and beyond the brain, ranging from the cellular level [15] and action–perception loops [16], to psychiatry [17], [18], [19], [20], [21], [22], psychology [14], [23], [24], [25] and embodied cognitive science [26], through to evolutionary dynamics (cf. [7], [27], [28], [29]) and, recently, to intersubjective and sociocultural dynamics [30], [31], [32], [33].

The FEP has recently been leveraged to furnish a fully generalisable metatheory for adaptive behaviour in sentient systems across spatial and temporal scales, called *variational neuroethology* (VNE) [4]. VNE synthesises the FEP with Tinbergen's [34] four levels of explanation in biology (i.e., adaptation, phylogeny, ontogeny, and mechanism) to provide a systematic guide for theorising and research in the sciences of life and mind. As a metatheory, VNE comprises two principal components: a multiscale theoretical ontology for living systems based on a recursively nested formulation of Markov blankets (MBs); and a multidisciplinary research heuristic in the biological and cognitive sciences [35]. In their response to peer commentaries, Ramstead and colleagues [4] address the promise and limitations of VNE as a heuristic for scientific inquiry, and review its scope as a research programme. This paper will complement this discussion by concentrating on criticisms of VNE as a *multiscale ontology*.

To date, VNE has only provided a *principled method* of analysing nested and mutually constraining biological systems and their complex adaptive dynamics across spatiotemporal scales [4], [35]. It has yet to offer a way to individuate systems at scales beyond that of the organism acting in its environment; e.g., large-scale ensembles such as societies or ecosystems that realise free energy bounding dynamics. This issue was cogently articulated by Bruineberg and Hesp [36] and Kirmayer [37] in their critique of VNE. They asked whether the MB formalism leveraged by VNE to individuate systems is adequate for modelling phenomena at scales beyond those of a single organism (e.g., sociocultural dynamics); since phenomena at these scales may be too transient or not sufficiently robust to license the MB formalism (which is defined in terms of conditional independencies in weakly mixing random dynamical systems). Could this reflect a fundamental distinction between the FEP as an explanatory principle for clearly bounded, ergodic, biological systems, and the greater, complementary forces at play that constrain complex adaptive systems in general (including groups of organisms and their ecological niches)? Should we restrict the FEP to the level of the organism, and then explore how this model connects meaningfully to other key concepts in evolutionary systems theory about the agent-niche relation?

The aim of this paper is to address the challenges faced by VNE with regard to dynamics that obtain for large-scale ensembles. Specifically, we review a framework for modelling *complex adaptive systems* for multiscale free energy bounding organism–niche dynamics, thereby integrating the modelling strategies and heuristics of VNE with a broader perspective on the ecological nestedness of biotic systems. We extend VNE beyond the action–perception loops of individual organisms (i.e., active inference, [38], [39]) by appealing to the *variational approach to niche construction* (VANC) [40] to explain the dynamics of coupled systems constituted by organisms and their ecological niche. We suggest that the statistical robustness of living systems is inherited, in part, from their eco-niches, as niches help coordinate dynamical patterns across larger spatiotemporal scales. We call this approach *variational ecology* (VE), which subsumes VNE and the VANC.^2^ When applied to cultural animals such as humans, VE has the important consequence of allowing us to formulate not just a physics of individual minds, but also a physics of *interacting minds* across spatial and temporal scales – a *physics of sentient systems* that range from cells to societies.

## 1 The variational (free energy) formulation

The free energy formulation appeals to a *statistical conception of life*. It rests on the fact that on average and over time, living systems endure as bounded, self-organising partitions of dynamical systems. In other words, organisms appear to counter dissipative environmental perturbations by resisting the weathering effects of entropic decay dictated by the fluctuation theorems that hold at nonequilibrium steady-state [41], [42]. Technically, biological systems revisit the same set of characteristic states that constitute a *random dynamical attractor*. This attracting set means they have properties that can be measured (i.e., they are locally ergodic). Put another way, an organism possesses an attracting set of states that it tends to occupy with a much higher frequency than others. The FEP provides a formal description of how organisms resist entropic erosion and maintain themselves within their phenotypic bounds. More exactly, it describes the dynamics they must exhibit, if they possess characteristic or attracting states.

For an organism to exist as a bounded system means that it must be able to maintain itself as a whole. By definition, for a system to exist at all, it must evince a robust form of *conditional independence* with respect to external (non-systemic) states. The variational framework addresses the individuation of relevant systems by operationalising the notion of conditional independence using the *Markov blanket* (MB) *formalism*. For a set of states to be enshrouded by a MB means that the *dynamics* of that system induce a *statistical partition* of its states [3], [4], [43]. A MB is the set of states that statistically isolates (insulates) *internal* (systemic) from *external* (non-systemic) states, such that changes in internal states are mediated by the states of the MB. The MB itself can be partitioned into *active* and *sensory* states, which are defined by the following relations: internal states do not influence sensory states, and external states do not influence active states.

Now, we should note that the terms ‘active’ and ‘sensory’ are potentially misleading. They are only meant to capture relations of *statistical dependence* between random variables. This will be crucial to our argument below, as things that we would not readily describe as literally acting or sensing in any meaningful sense can still be captured with this formalism, since it entails only a statistical enshrouding of systemic states from external ones, and the systematic statistical partition of the whole organism–niche system [4].

### 1.1 Active inference and generative models

To keep the MB in play is not a trivial matter. The FEP explains the emergence and maintenance of this existential boundary. Under the FEP, the Markov boundary is dynamically enabled by *adaptive action*. The idea behind the FEP is that the *active behaviour* of organisms maintains them in states of viable, adaptive coupling with their ecological niche. *Active inference* is the process whereby living systems act on the world – and update their internal states – so as to embody or encode the statistical structure of their local environment, leading to the *adaptive control of behaviour* [38], [44]. In other words, over time and on average, biological systems come to fit their environment, via active optimisation procedures; e.g., action, perception, and learning [45]. From a developmental perspective, this can be cast as phenotypic accommodation and developmental plasticity [46], [47].

Crucially, this functionalist interpretation is underwritten by the gradient flows implied by the existence of a random dynamical attractor (and implicit ergodicity). This is fairly straightforward to show using either a path integral or Fokker Planck formulation of density dynamics; in which the flow of states must climb probability gradients to counter the dispersive effects of random fluctuations. When we put the MB in play, the same gradient flows reveal an (en)active and adaptive interpretation of active states, which are informed by, and only by, sensory and internal states. This follows because the flows of active and internal states are special, in the sense that their flow does not depend upon external states. These structured dependencies, which necessarily follow from the existence of random dynamical attractors with MBs, lead to active inference.

**Fig. 1:**
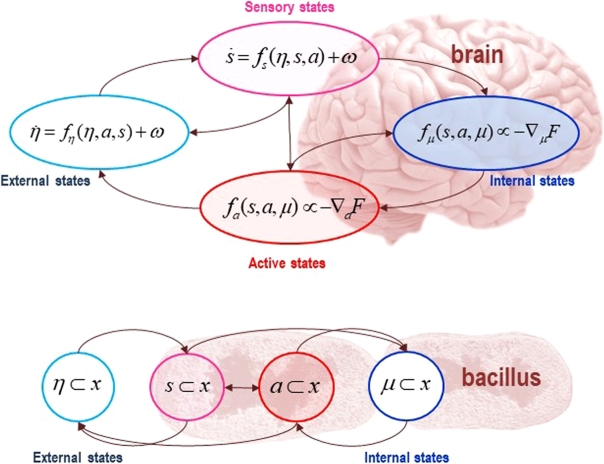
*The Markov blanket*. These schematics illustrate the partition of states into internal (***μ***) and hidden or external states (***η***) that are separated by a MB – comprising sensory (***s***) and active states (***a***). The upper panel shows this partition as it would be applied to action and perception in the brain; where active and internal states minimise a free energy functional of sensory states. The ensuing self-organisation of internal states then corresponds to perception, while action couples brain states back to external states. The lower panel shows exactly the same dependencies but rearranged so that the internal states are associated with the intracellular states of a cell, while the sensory states become the surface states of the cell membrane overlying active states (e.g., the actin filaments of the cytoskeleton).

In active inference, *active* and *internal states* of the organism's *MB* can always be expressed as minimising or *bounding* a quantity called *variational free energy* (see Fig. 1). We can cast variational free energy as the disattunement between the statistical structure of the ecological niche and that of the organism (i.e., its phenotype and behavioural dynamics) [26], [48]. Technically, variational free energy is a *proxy* for a quantity called surprise (a.k.a. *surprisal*), which reflects the improbability of finding an organism in some sensory state (technically, surprise is the self-information or negative log probability of sensory samples encountered by an agent) [2]. The organism cannot evaluate this quantity directly. Instead, it (or rather, its behaviour or dynamics) bounds a *proxy quantity*, which is a variational bound on surprise, in the sense that surprise can never be greater than this bound. This is variational free energy and is exactly the same quantity used in machine learning and variational Bayes (often referred to as an *evidence bound*). While surprise depends only on states of the world, variational free energy depends on a Bayesian belief or probability density that is encoded by its internal states. Through active inference, the probability densities entailed by organismic (internal) states tune themselves to effectively infer the process by which sensory states were generated; i.e., the dynamics of external, unobserved states that cause fluctuations in sensory states and are hidden behind the MB.

Under the FEP, to function as a *control system* for the organism, and ensure it remains within phenotypic states, the dynamics (i.e., *adaptive behaviour*) of the organism entails a *statistical model* (that is, a *generative* model) of *itself acting in its ecological niche*. In this framework, a generative model is a probabilistic mapping from causes in the environment (including, crucially, the actions of the organism itself) to sensory states (observations); while the generative process is the actual causal structure that generates sensed consequences from external causes that are hidden behind the MB [49]. This inference is a necessary consequence of gradient flows that minimise variational free energy. This follows because minimising variational free energy is – mathematically – the same as maximising the *evidence bound* in machine learning and Bayesian statistics. In this instance, the evidence is for a generative model entailed by the internal states of a creature – technically, if the internal states parameterise a posterior density over external states, then internal (and active) states will maximise the evidence for a generative model of external, niche dynamics. Active inference therefore means that the *dynamics of living systems entails a generative model*. *The optimisation of this model corresponds to bounding or minimising variational free energy*, which can be summarised as *self-evidencing* [50]. This notion of self-evidencing places the MB centre stage as both an evidentiary and existential boundary.

In summary, active inference can be cast as the process that confirms and updates evidence for the statistical (generative) model that a living system entails and enacts *in existing* – thereby producing evidence for its own existence [50]. This neatly covers action and perception in a folk psychology sense; because internal states cannot, in themselves, change sensory states, but they can optimise the probabilistic explanations (i.e. posterior probability distributions or Bayesian beliefs) for sensory impressions by minimising variational free energy. This has all the look and feel of perception. Conversely, active states cannot change posterior beliefs but they can change sensory states; either directly or vicariously via external states. This corresponds to action, which is informed by perception. In short, the MB will actively seek evidence for its own existence. Over time, the states of the organism (e.g., the brain) come to encode the statistical structure of causal regularities in the world, and underwrite the generative model – the *behavioural control system* (cf. [51], [52]) that regulates patterns of interaction with the environment. This sort of slow perception can be regarded as learning and is generally associated with plasticity of a developmental or experience-dependent sort in internal states that comprise internal structure and connectivity.

Two recent extensions of the free energy formulation will be the focus of our attention. These are variational neuroethology (VNE) [4] and the variational approach to niche construction (VANC) [40]. VNE and the VANC are about enabling the application of the FEP to phenomena within and beyond the brain. These approaches hold the promise of extending the variational (free energy) approach to the dynamics of sentient systems (i.e., systems with sensory states) across spatial and temporal scales.

## 2 Variational neuroethology

VNE provides a framework for modelling the dynamics of sentient systems across the spatiotemporal scales they manifest and an explanation or description of their self-organisation. Formally, VNE provides a principled way of *scaling up* active inference over: (i) ensembles of MBs; and (ii) MBs of MBs. This allows us to formulate an integrative multiscale dynamics that link the partial dynamics of phenomena at each scale (also see [53]). In what follows, we speak to the multiscale aspects of MBs and then turn to ensembles of MBs that are coupled to each other.

### 2.1 Multiscale levels of analysis

VNE synthesises the process theory derived from the FEP (active inference) with Tinbergen's seminal four questions in biology (i.e., adaptation, phylogeny, ontogeny, and mechanism) to propose a heuristic guide to research in the sciences of life and mind. Integrating these two paradigms allows substantive insights drawn from one to inform and constrain models and research in the other – the FEP is a non-substantive principle that can be applied to any biological system in general (much like Hamilton's principle of least action), while Tinbergen's framework can provide substantive explanations for biological phenomena drawn from four complementary levels of analysis [54]. This heuristic has already inspired an interdisciplinary theory of the human brain called the Hierarchically Mechanistic Mind ([4], Badcock, P.B., Friston, K.J., Ploeger, A., Hohwy, J., and Ramstead, M.J., in review, [23]). The HMM rests on the idea that the brain is a hierarchically structured, self-organising system that has been sculpted by natural selection [23], [25], [55], [56]. It suggests that the dynamics, structure, and function of the human brain instantiate an embodied, situated, *complex adaptive system* that actively minimises free energy by generating adaptive action–perception cycles via recursive interactions between hierarchically nested, functionally differentiated subsystems ([23] Badcock, P.B., Friston, K.J., and Ramstead, M.J.D. (this issue)). By synthesising the FEP with Tinbergen's four levels of analysis, this perspective can be reduced to four complementary research questions – What is the adaptive function of a phenotypic trait? How does it emerge from circular interactions between phylogenetic (resp. intergenerational), ontogenetic and mechanistic processes? In what ways does it instantiate the FEP? And how does it manifest in hierarchical neural dynamics? This research heuristic has already been leveraged to develop a new evidence-based theory of our capacity for depression [25].

**Fig. 2:**
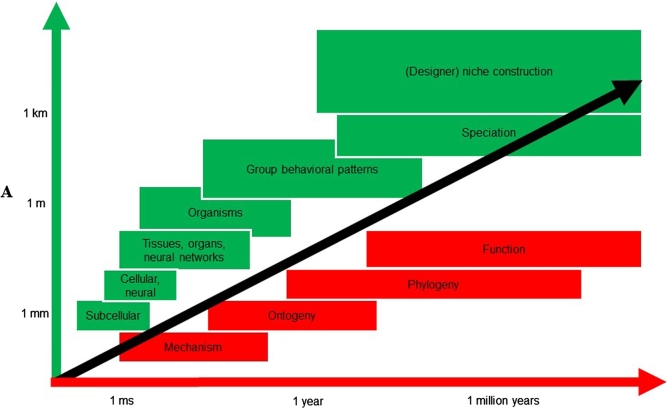
*Explanatory scope of variational approach*. Variational neuroethology leverages the FEP to explain the adaptive free energy bounding dynamics of living systems across spatial and temporal scales. Here we indicate some of the scales at which these dynamics unfold. Given the intrinsic correlation between spatial and temporal scales, the phase space described here is populated mostly along its diagonal. Adapted from [92].

On the other hand, VNE entails a *multiscale ontology* for living systems as well. This ontology extends the MB formalism as a method of individuating systems under the FEP. As discussed above, a MB is a set of states that separates a system (a set of *internal* states) from *external*, non-systemic ones, which operationalises the idea that systems only exists *per se* if they are endowed with a robust form of conditional independence. According to VNE, the dynamics of living systems can be cast as active inference over *recursively nested* MBs (see Fig. 2). Indeed, the MB ontology can be reiterated recursively, such that the MBs at any one scale are composed in turn of MBs at the scale above and below – which are also made of MBs, and so on, all the way up, and all the way down [43].

**Fig. 3:**
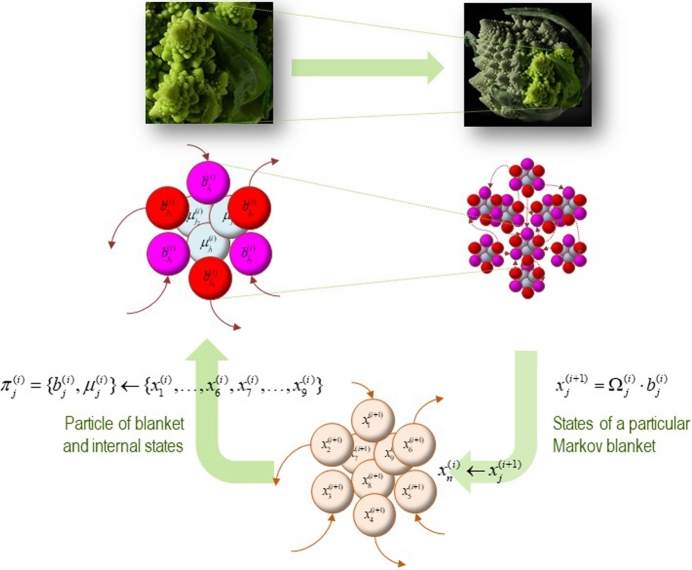
*Blankets of blankets*. This schematic figure illustrates the recursive architecture by which successively larger (and slower) scale dynamics arise from subordinate levels. Starting at the bottom of the figure (lower panel) we can consider an ensemble of vector states (here nine). The conditional dependencies among these vector states then define a particular partition of into *particles* (upper panels). Crucially, this partition equips each particle with a bipartition into blanket and internal states, where blanket states comprise active (red) and sensory states (magenta). The behaviour of each particle can now be summarised in terms of (slow) eigenmodes or mixtures of its blanket states to produce vector states at the next level or scale. These constitute an ensemble of vector states and the process starts again. The upper panels illustrate the bipartition for a single particle (left panel) and an ensemble of particles; i.e., the particular partition per se (right panel). The insets on top illustrate the implicit self-similarity in moving from one scale to the next using. In this figure, Ω ⋅ *b* denotes a linear mixture of blanket states specified by the principal eigenvectors of their Jacobian. Because the corresponding eigenvalues play the role of Lyapunov exponents, the resulting mixtures correspond to slow or unstable dynamical modes of activity.

The key notion here is that in moving from one level or scale of dynamics to the next, things not only get bigger, they also get much slower. The basic idea is that the states at one scale constitute microscopic states that can be partitioned into an ensemble of MBs. To move to the higher scale, one treats each MB as an entity (e.g., particle) and summarises its dynamics with mixtures of blanket states that fluctuate slowly. In this multiscale setting, a (effective) state at any scale becomes the expression of an eigenmode of blanket states; namely, the principal eigenvectors of their Jacobian (i.e., rate of change of flow with respect to state). These mixtures are formally identical to *order parameters* in synergetics that reflect the amplitude of slow, unstable eigenmodes [57]. In terms of centre manifold theory, they correspond to solutions on the slow (unstable or centre) manifold [35], [58]. In short, the MB of a system (or particle), at any scale, constitutes an ensemble whose order parameters subtend blanket or internal states at the scale above. Note that the constituent (microscopic) states of an ensemble are always blanket states, although their order parameters could be blanket states or internal states at the (microscopic) scale above. This follows from the fact that the only states ‘that matter’ are those that influence other (blanket) states. Effectively, all we are doing here is applying the (en)slaving principle, or centre manifold theorem [57], recursively to MBs of MBs. Fig. 3 provides a schematic illustration of this recursive decomposition.

In this multiscale framework, active inference is inherently a group activity. That is, the entire ensemble of nested MBs are bound, enslaved and constrained by dynamics at higher scales, while the lower (microscopic) scales furnish the (macroscopic) states at any given level. This construction evinces exactly the same circular causality that underlies synergetics [57], [59]; however, here it is generalised to a recursive hierarchy of scales – i.e., the hierarchical composition of blankets of blankets. Intuitively, the dynamics at one scale provide constraints (technically, establish probability gradients) on dynamics at other scales. Active inference destroys free energy gradients at each scale,^3^ under the guidance or control of a generative model at the scale above. This guidance is exerted through influences on sensory states, where circular causality means that the action of any MB in an ensemble of MBs could be involved in sensing, action or perception; depending upon its role at the superordinate scale; i.e., has a sensory, active or internal state at the level above. Please see [43] for a worked example using simulations of biological self-assembly.

**Fig. 4:**
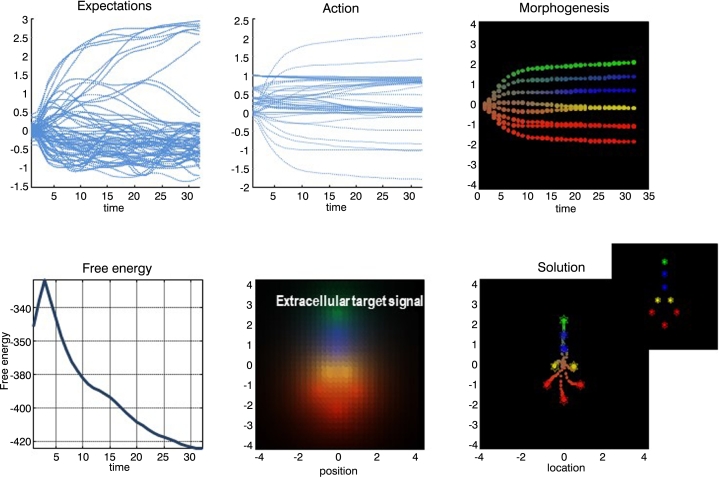
*Self-assembly and active inference*. This figure shows the results of a simulation of morphogenesis under active inference reported in [70]. This simulation used a gradient descent on variational free energy using a simple ensemble of eight cells; each of which had the same (pluripotential) generative model. This generative model predicted what each cell would sense and signal (chemotactically) for any given location in a ‘target morphology’ (lower middle panel – extracellular target signal; in other words, what each agent would expect to sense if it were at a particular location). By actively moving around, all the cells minimised their variational free energy (i.e., surprise) by inferring where they were, in relation to others. Because variational free energy is an extensive quantity, the free energy minimising arrangement of the ensemble is the target morphology. In other words, every cell has to ‘find its place’, at which point each cell minimises its own surprise about the signals it senses (because it knows its place) and the ensemble minimises the total free energy. The upper panels show the time courses of expectations about its place in the morphology (upper left), the associated active states mediating migration and signal expression (upper middle) and the resulting trajectories; projected onto the first (vertical) direction – and colour-coded to show differentiation (upper right). These trajectories progressively minimise total free energy (lower left panel). The lower right panel shows the ensuing configuration. Here, the trajectory is shown in small circles (for each time step). The insert corresponds to the target configuration. Please see [70] for further details.

This may sound a little abstract; however, imagine you are an employee as an institution, where you transact your (microscopic) affairs with other personnel to self-evidence your prior beliefs that you are ‘good at your job’. This would entail responding to corporate or institutional goals that emerge collectively (i.e., an implicit generative model at the macroscopic level). Note that your job may be homologous to an internal state at the institutional (macroscopic) level – relating only to other employees. Alternatively, you could be working on reception (i.e., a sensory state) or issuing press releases (i.e., an active state). Another example of multiscale self-organisation is provided in Fig. 4 to illustrate a less anthropomorphic form of self-assembly at the cellular and molecular level. In this example, the extensive nature of variational free energy is laid bare: each system or agent that comprises the ensemble shares the same generative model. This means that the total free energy is composed in exactly the same way statisticians would accumulate statistical evidence through Bayesian belief updating with each new source of evidence.^4^ The twist here is that the (sensory) evidence for each agent's model is generated by another agent. In short, multiscale ensembles that endure, in an ergodic sense, dissolve free energy gradients, thereby integrating dynamics within and between scales. This brings us to questions about coupling among MBs within a particular scale, which will be our focus for the remainder of the paper.

### 2.2 Ensembles of MBs

In what follows, we look more closely at the partition of states into an ensemble of MBs – and what this means for self-organisation at any scale of dynamics. A crucial point, which enables the integration of ensemble dynamics into *adaptive behaviour*, is that variational free energy is an extensive quantity; in other words, it increases with the size or compass of the system in question. In the context of an ensemble of MBs, this means we can add the free energy of each agent or particle to describe the behaviour of the ensemble (see Fig. 4).

Applying the MB formalism to sentient systems is intuitive when modelling systems whose conditional independence is maintained via literal sensorimotor loops, where the interaction between active and sensory states ranges over relatively small spatiotemporal scales. Note that every leap upward in the nested hierarchy implies a concomitant increase in spatial and temporal scale (see Fig. 3), which in some cases entails an increase in the distances among the states of the system, as well as a decreased rate of change and (Bayesian) optimisation. As we ascend spatiotemporal scales, it becomes increasingly unclear how the Markov boundaries are implemented. Indeed, the justification for the use of the MB formalism to describe living systems rests on the observation that if the coupling among an ensemble of dynamical subsystems is mediated by short-range forces, then the states of those remote subsystems must be conditionally independent [3]. In summary, despite providing a framework to integrate dynamics across scales – and ensemble dynamics within a scale – VNE does not tell us how large scale ensembles self-organise so as to form higher-order MBs. In the next section, we address this issue; namely, how to draw MBs for large-scale systems like ecological niches, social ensembles, and cultural dynamics, drawing on the notion of *affordances* from the skilled intentionality framework [48], [60].

## 3 The variational approach to niche construction and the ontology of affordances

### 3.1 The variational approach to niche construction

Niche construction theory in evolutionary biology (e.g., [61], [62], [63], [64]) argues that via their bioregulatory behaviour, living organisms (explicitly and implicitly) modify their environment, so as to steer their evolutionary trajectory, and that of other species. Arguably, then, niche construction is on a par with natural selection as a *bona fide* evolutionary force. Niche construction can be understood in two ways: in terms of selective niche construction (SNC), or in terms of developmental niche construction (DNC) [64]. SNC refers to changes to the ecological niche induced by the action of organisms, and by which living systems come to modify selection pressures on themselves and other species that inhabit the niche. SNC operates on a phylogenetic time scale, and involves processes like ecological and cultural inheritance [65], [66], where evolutionarily significant components of an environment are passed on from one generation to another; e.g., the remains of beaver dams are reconstructed by beavers, while the concept of a ‘canoe’ is passed down generations in the form of ‘cognitive gadgets’ [67]. DNC refers to the production in ontogeny of *exogenetic resources* by organisms themselves; in order to change developmental inputs, and secure the reliable and flexible reproduction of the individual life cycle [64]. DNC operates on the scale of development, learning, and action–perception cycles. The set of exogenetic resources that it optimise's include evolved loops of adaptive behaviour (e.g., grooming, parental care) and physical changes to the niche itself [68].

As an example of niche construction outcome in humans, consider improvised ‘desire paths’; e.g., a dirt trail in the park carved out by recurrent actions of agents in the neighbourhood. From the point of view of the FEP, such a path can function as an exogenetic resource. It encodes precise (high certainty, reliable) information about the fact that the end of the park is at the end of the trail. An agent can rely on this information to navigate the park efficiently, without having to know the layout of the neighbourhood. While crossing a park might not be as essential a behaviour as grooming, from the point of view of the FEP, finding oneself in expected sensory states certainly is. Indeed, exogenetic resources of the niche such as desire paths can function as reliable indicators of surprise- and ambiguity-resolving actions, which, under the statistical conception of the phenotype entailed by the FEP, is crucial for maintaining the agent's continued existence. In short, the collective behaviour of an ensemble of agents provides a form of semiotics or a set of *possibilities for engagement with the niche* (a.k.a. *affordances*) that become relevant from the point of view of the needs and concerns of any single agent. So how can a variational ecology shed light on the role of affordance in selecting adaptive actions that emerge from ensemble dynamics?

**Fig. 5:**
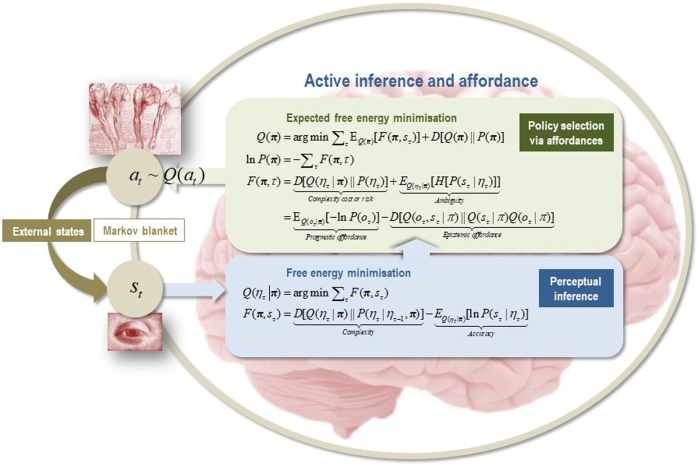
*Bayesian mechanics and active inference*. This graphic summarises the belief updating scheme in the minimisation of variational free energy and expected free energy [38], [45], [93]. In the first step (circles on the left), discrete actions solicit a sensory outcome (i.e., in the parlance of the SIF, a *solicitation*) used to form approximate posterior beliefs about states of the world. This belief updating involves the minimisation of free energy under a set of plausible policies (blue panel – Perceptual inference). Note that free energy *F*(***π***,*s*) includes Markovian dependencies among hidden states. This reflects the fact that the generative model is a Markov decision process. In the second step (green panel – Policy selection), the approximate posterior beliefs from the first step are used to evaluate *expected* free energy *F*(***π***,*τ*) and subsequent beliefs about action. These beliefs correspond to the epistemic and pragmatic *affordances* that underwrite policy selection. Note that the free energy *per se* is a function of sensory states, given a policy. In contrast, the expected free energy is a function of the policy. The construct of *affordance* in active inference corresponds to *inferences about action* on the environment, which are selected in terms of competing policies via the minimisation of expected free energy. The variables in this figure correspond to those in Fig. 1. Here, a policy ***π*** comprises a sequence of actions; the expression *Q*(*η*|***π***) denotes beliefs about hidden states given a particular policy; and *Q*(***π***) denotes posterior beliefs about the policy that is currently being pursued by the agent. Free energy is the difference between *complexity* and *accuracy*, while expected free energy can be decomposed into expected complexity (i.e., complexity cost or *risk*) and expected inaccuracy (i.e., *ambiguity*). Risk can be regarded as the (KL) divergence (*D*) between beliefs about future states under a particular policy and prior preferences about states. Ambiguity denotes the loss of a definitive mapping between external states and observed sensory states (quantified as entropy, *H*). Alternatively, expected free energy can be decomposed into *epistemic* and *pragmatic affordance*. Posterior beliefs about policies depend on their expected free energy. Crucially, these posterior beliefs include the free energy evaluated during perceptual inference. This has several interesting consequences from our perspective. This construction means that the agent has to infer the policy that it is currently pursuing and verify its predictions in light of sensory evidence. This is possible because the beliefs about actions that are encoded by internal states are distinct from the active states of the agent's MB. Free energy *per se* provides evidence that a particular policy is being pursued. In this scheme, agents (will appear to) entertain beliefs about their own behaviour, endowing them with what is defined as *intentionality* of goal directed behaviour under active inference. In effect, this enables agents to author their own sensorium in a fashion that has close connections with niche construction: see main text and [48]. See [94] for technical discussion. Figure from [95].

Selecting adaptive actions requires an organism to evaluate *expected free energy* under possible action policies [69]. Expected free energy can be expressed in different ways; e.g., as expected energy minus entropy, or as a mixture of epistemic and pragmatic value (see Fig. 5). With respect to our purposes here, expected free energy can be expressed as the *expected cost* of an outcome, given a certain action, plus the expected *ambiguity* of the outcome (cf. [70]). *Expected cost* corresponds to the *discrepancy* between outcomes conditional on a given action, and expectations or preferences about outcomes (technically, the Kullback–Leibler divergence between the two beliefs). Evaluating expected cost enables the selection of actions that bring about sensory states (observations) expected by the agent – i.e., those predicted by its generative model, and by the same token, those that once brought about, have the least deleterious or ‘costly’ consequences with regard to their surprisal. In turn, the ambiguity reflects an agent's expectations about the uncertainty of outcomes, dependent upon causes in the world.

This is where the variational approach to niche construction (VANC) comes in. VANC exploits the expression of expected free energy as expected cost plus ambiguity, to propose that agents *upload*, as it were, much of the leg work in computing expected free energy to their self-tailored, constructed environment. More precisely, it argues that *niche construction* can be cast as the collective activity by which organisms act on their material environment to create unambiguous structure, which can be leveraged (via active inference). Lasting changes to the niche capture the fact that environmental cues function as an unambiguous indicator of the affordance of action possibilities. These can be cast in terms of *epistemic resources* that flag those actions that resolve the ambiguity associated with future observations [71], while conforming to the (prior) preferences of the organism entailed by its generative model.

VANC casts DNC as the joint optimisation of the niche – and its constituent ensemble of agents – over ontogeny through dense histories of active inference, and casts SNC as Bayesian model selection [29], [40]. By engaging ecologically inherited exogenetic resources of the niche, living organisms – especially those, like humans, that depend on large-scale coordination – can reliably track, predict, and model the unfolding of causal regularities at and across large spatiotemporal scales [54]. We will argue that this is so because niches themselves (including the organisms and their material setting) track those regularities. For instance, a complex system of agriculture that employs irrigation techniques enables groups of humans to smoothly cope with climate fluctuations that could otherwise jeopardise food production. In Section 4, we explain how the existence of higher-order, large-scale human ensembles depend on specific exogenetic resources of the niche – namely, *epistemic* resources of the kind just discussed – and how this emerges from ensembles of agents engaging in active inference.

### 3.2 The ontology of affordances under the variational approach

The skilled intentionality framework (SIF) [60] provides an account of the origin of *intentionality* or directed purposiveness in cognitive systems. We appeal to the SIF in offering an account of how to draw higher-order MBs; namely, by mobilising the notion of *affordances*. The SIF recasts cognition as the engagement by organisms of the affordances that make up their local niche, thereby providing a real-time dynamics for the engagement of exogenetic (epistemic) resources that the niche affords, and enabling the stabilisation of the local niche.

What are affordances, and how are they to be interpreted under the free energy formulation? The SIF defines affordances as possibilities for engagement that obtain between a set of abilities at an organism's disposal and relevant or salient features of the material environment [33], [48], [72], [73]. Here, ‘engagement’ refers to structured, skillful patterns of action and perception^5^ – what we have covered under the rubric of *active inference*. In effect, the SIF provides an organism-centred dynamics that explains, concretely, what it means for an agent or group of agents and their ecological niche to engage in active inference and niche construction. Moreover, it does this by considering explicitly how the ecological niche is disclosed to the organism – as a *field of affordances*.

The SIF tells us what is special about living systems in terms of their being directed towards meaningful worlds (or strictly speaking, outcomes). Namely, it points out something special about the way living systems self-organise. The dynamics of most (non-living) self-organising systems emerge and stabilise around an *energy gradient*, which those same dynamics then typically resolve or consume; e.g., as a lightning bolt strikes, the charge gradient around which it organised dissipates. Unlike other self-organising systems, living organisms are unique in that they actively generate and maintain the gradients that sustain them, through adaptive actions. In other words, an organism's self-evidencing underwrites self-organisation and the very ergodicity upon which both rest [48], [74].

What are the gradients around which living systems organise? The variational framework suggests that these gradients are *variational free energy gradients* resolved through active inference. Consistent with the FEP, under the SIF, *affordances* are cast as *expected free energy gradients* or differences. These differences are in the expected free energy associated with the repertoire of actions or abilities available to an organism under its generative model and the learned niche [33], [48]. In formulations of active inference for generative models of discrete states (i.e., Markov decision processes), these abilities correspond to the policies and their affordance is quantified in terms of the expected free energy under each policy. Given this equivalence, affordance can be decomposed into *complexity cost* and *ambiguity* (as in Fig. 5). Alternatively, by rearranging its terms, expected free energy can be expressed in terms of *epistemic* (i.e., intrinsic) and *pragmatic* (i.e., extrinsic) affordance (see [71], [75] for details). The ensuing variational formulation of affordances uses the path integral of free energy from the current point in time to a future time point, where the only difference between expected free energy and free energy *per se* is that sensory states have yet to be realised. This means the *expectation* in *expected* free energy is over sensory states in the future, based upon posterior beliefs informed by sensory states in the past. Put simply, the best action is the next action that belongs to the policy (i.e., sequence of actions) with the greatest affordance – or the least expected free energy. This is formally related to Hamilton's principle of least Action^6^ that translates here into a variational principle of greatest ‘Affordance’.

For organisms to engage the affordances offered by their niche is the variational ‘tissue’ that connects dynamics to the niche in which those organisms exist. The expected free energy of each policy can be cast as constituting the set of affordances that an organism can entertain. Action and policy selection integrate ensemble dynamics, by minimising expected free energy directly and learning the affordances on offer from the niche by selecting courses of action (i.e. policies) with the greatest epistemic affordance. This has close relationships with intrinsic motivation and exploration in ethology [76], [77], [78], [79] where, perhaps, the most important exploration is “what can I do with my body?” (i.e., the body as niche). This is most clearly seen in development and neurorobotics [78]. Things get more interesting when we appreciate that the niche itself is subject to exactly the same normative principles. In other words, as each agent is trying to learn about and infer its niche, the niche – through the collective inferences, actions and material artifacts of its constituent agents – appears to learn about and predict the behaviour of each agent. This must be true, because each MB that comprises the eco-niche is itself trying to minimise variational free energy. Heuristically, this means that because every agent is trying to predict their niche, they collectively shape their field of affordances in such a way that their niche appears to infer the behaviour of its agents and therefore becomes inherently more predictable (i.e., less ambiguous).

This prompts a revision of the ontology of affordances under the SIF [48]. The ‘field of affordances’ is the set of affordances that solicit the organism at a given time. This field is constituted by expected free energy gradients that are induced by the entire ensemble. The engagement of the niche by the organism then corresponds to active inference; in the sense that these dynamics are simply the resolution of a local free energy landscape – a path of least (Hamiltonian) Action over the field of affordances. ‘Solicitations’ are those affordances that effectively engage the organism in action–perception loops at a given time. The ‘landscape of affordances’ is thus the set of affordances available in a niche at a given time. On this view, the landscape of affordances is a product of inference about “what would happen if I did that?” [79]. Affordance is therefore an attribute of active, if counterfactual, engagement with the niche. Yet at the same time, it is a statement about the learned (and therefore lived) world.

The picture that emerges by integrating a variational approach to niche construction with the ontology of affordances could be summarised as follows. When you select actions with the greatest affordance, you learn about your niche. However, your niche comprises other MBs that must be learning about you. These can be other ‘creatures like you’, ‘cognitive gadgets’, and ‘desire paths’, and so on. In other words, your action on the environment constitutes sensory evidence for a niche (i.e., landscape of affordances) that is trying to model you, while the niche acts on you via sensory impressions. From the perspective of some ‘Godhead’ looking down on your niche, a self-organisation would emerge at a higher spatiotemporal scale – that itself looks exactly like a self-organising, free energy minimising process. This has to be the case if your niche and all of its constituents attain some form of ergodicity, in virtue of the FEP. In other words, the network of conspecifics, their ‘desire paths’, and ‘cognitive gadgets’ would look like the internal states surrounded by a MB, separating your niche from another. We now develop this argument in the final section.

## 4 Variational ecology: a physics of shared minds

How can we describe the sort of relationships that induce conditional independence among remote subsystems of ensembles (e.g., other animals as part of a larger niche)? Can we make sense of the self-organisation of large-scale systems using MBs, to constitute robust and enduring sets of conditionally independent subsystems? How are MBs implemented at higher scales, and how can we define the relations among their states?

**Fig. 6:**
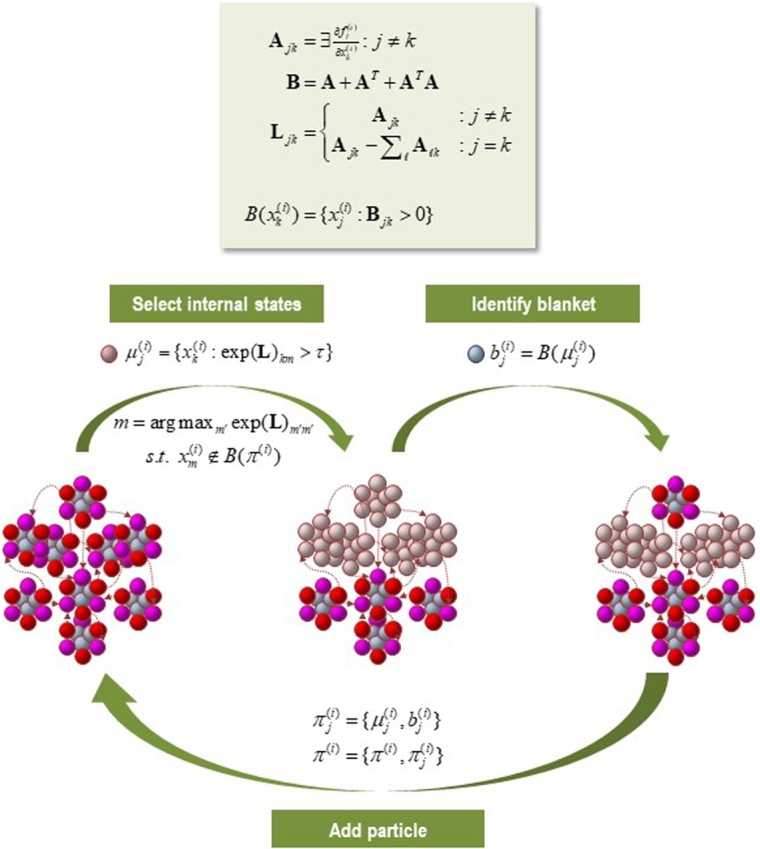
*A particular partition*. This schematic figure illustrates a partition of vectors states (small coloured balls) into particles (comprising nine vectors), where each particle (*π*) has six blanket states (red and magenta for active and sensory states respectively) and three internal states (cyan). The upper panel summarises the operators used to create a particular partition. We start by forming an adjacency matrix that characterises the coupling between different vectors states. In this example, the adjacency matrix is based upon the Jacobian and implicitly the flow of vector states. The resulting adjacency matrix defines a MB forming matrix (**B**), which identifies the children, parents, and parents of the children. The same adjacency matrix is used to form a graph Laplacian (**L**) that is used to define neighbouring (i.e., coupled) internal states. One first identifies a set of internal states using the graph Laplacian. Here, the *j*-th subset of internal states at the level *i* are chosen based upon dense coupling with the vector state with the largest graph Laplacian. Closely coupled internal states are then selected from the columns of the graph Laplacian that exceed some threshold. In practice, the examples used later specify the number of internal states desired for each level of the hierarchical decomposition. Having identified a new set of internal states (that are not members of any particle that has been identified so far) its MB is recovered using the MB forming matrix. The internal and blanket states then constitute a new particle, which is added to the list of particles identified. This procedure is repeated until all vector states have been accounted for. In the example here, we have already identified four particles and the procedure adds a fifth (top) particle to the list of particles; thereby accounting for nine of the remaining vector states.

The internal states of a MB can be separated by a great deal of distance, while retaining some form of dependency. When looking at phenomena unfolding across longer temporal scales, imagining internal states as *physically isolated in a literal sense* from external states becomes conceptually constraining. Two states clearly do not need to be (and as a matter of fact cannot be) in direct contact to be part of the same system; e.g., nodes of the Internet, soldiers in a battalion. What matters is the *statistical relationship* between states; i.e., to form a MB, the right kinds of statistical relations and partitions need to be in play. In other words, ensembles sharing the same MB, whether cells or organisms, need not be ‘spatially’ compartmentalised, but ought to be ‘statistically’ or ‘behaviourally’ segregated; that is, some *recurrent patterns of behaviours* should govern the way different sets of internal states maintain their *conditional independence*. Practically speaking, the only thing one needs to partition states into an ensemble of MBs is their adjacency matrix. This matrix (from graph theory) encodes directed dependencies in a weighted or unweighted fashion. One can then identify a subset of internal states, their MB, and the resulting external states [3]. This process can be repeated by identifying a second set of internal states within the external states and continuing until all external states have been exhausted. Note that, to pursue this partition into an ensemble of MBs, one only needs the adjacency matrix describing ‘who is connected to whom’. This can easily be applied to differential equations describing evolutionary dynamics – or links in social media networks. See Fig. 6 for an illustrative example. The question then is to determine how the *active and sensory states* are manifest, where the partition of states is dispersed over high dimensions of abstract state spaces. In other words, we need to understand what sort of ‘behaviour’ their states exhibit.

### 4.1 Markov blankets and ensemble dynamics: active and sensory states

Under the MB formalism, sensory and active states are to be interpreted in their minimal sense; that is, as *instantiating statistical relations*. What it means for a system to have active states is for it to have states the dynamics of which change as a function of some systemic quantities (namely, as a function of internal and sensory states). In turn, what it means for something to have sensory states is for it to have states that change as a function of other systemic quantities (external and active states). On this view, both sensing and acting assume a statistical definition. Indeed, one could wonder about the difference between an agent sensing things in the world with a visual saccade and a photon hitting the retina of the agent. In the parlance of the MB formalism, whether a state counts as an active or a sensory state depends on whether or not it is influenced by internal and external states in the right way [3]. Thus, an active state is just one that is influenced by the system's defining states, and a sensory state is one that is not. Accordingly, a sensory state – at any level of description – might just mean a state that reliably covaries with, and conveys information about, some distal causes in the external system to which it is coupled (e.g., a receptionist taking calls on an external telephone line); and active states are just those states that enable the system to effect changes on the ‘outside’ (e.g., a public relations officer emitting press releases). An ecological niche could thus have sensory and active states (and thus, have a MB) in a sufficient and minimal statistical sense.

For a number of reasons, elements of the ontology of affordances are interesting candidates for the implementation of the sort of large scale behaviour under consideration. First, similar to the cell membrane, the sensory signals that actively engage an organism at a given time (i.e., *solicitations*) mediate the functional relationship between internal and external states of both the organism and its niche. In the parlance of the FEP, solicitations secure adaptive exchanges among the internal and external states by engaging the organism in loops of active inference that conform to internal expectations (e.g., they are perceived relative to the needs of the system), while enabling the learning of the causal structure of some external states (i.e., they are perceived relative to the actual causal structure). In this respect, similar to cell membranes, we can say that a solicitation “points both ways, to the environment and to the observer” [80]. In Fig. 1, this ‘pointing both ways’ is established in virtue of the circular causality induced by the MB and ensuing active inference. This brings affordances into play, in terms of the expected free energy attributed to different policies of courses of action on the environment (see Fig. 5).

Second, affordances, especially those enabled by epistemic resources, carry cultural knowledge that can be acquired in ontogeny [81], thereby securing the reproduction of adaptive patterned practices when transmitted across generations [40]. As such, affordances – especially the *cultural* affordances [33] that characterise a given local group – play a role in the coordination of the adaptive behaviour of members in a group over large spatial and temporal scales. Briefly, cultural affordances are the affordances with which human agents interact, and which depend on shared sets of cultural expectations, internalised through immersive practices [33]. Affordances thus allow for adaptive behavioural self-organisation among groups of agents, independent of their spatial proximity. In effect, cultural information encoded in the physical states of the ecological niche (in epistemic resources) enables the recognition and diffusion of epistemic affordances among groups over larger spatiotemporal scales (e.g., the intergenerational scale, via the passing on of affordances via cultural inheritance).

Third, defining a MB for niche systems involves the description of the conditional independence among the partitions of the system. The ontology of affordances, especially the layering it entails (e.g., organism and field, solicitation and landscape of affordances) can, in principle, be used to define the statistical compartments (partition) of the niche, and by the same token explain the conditional independence among the states of the niche.

Given these very reasons, we suggest that the *field of affordances* (and especially the solicitations that actually engage the agent at a given time) functions as the ‘surface’ that allows the niche to ‘sense’ agents via the agent's action on the niche [82]. The agent's actions when repeated over time encode regularities about niche-agent interactions via changes to the structure of the ecological niche. In virtue of the circular causality discussed above, this structure is determined by – and indeed, determines – the affordances that underlie each agent's action. The niche then ‘acts’ on the agent as it is sensed by the agent. We can say that an agent will be acted upon when engaging an affordance, as the affordance is a possible action to be selected, and the selection of which will entail changes in the agent. The action of the niche thus takes the form of ‘offering possibilities for engagement’. Crucially, a niche that would not offer a *variety* of possible engagements could not ‘act’ in any meaningful sense. The ‘active’ property of the niche rests on their ability to conform to the changing needs of the agent(s), that is, to solicit the agent by providing the right sort of sensory cues.

### 4.2 What is it that models, and what is modelled? – internal and external states

So far, we have discussed the relevant quantities to define the niche's MB. These are captured by the dynamics of the agent's field of solicitation, which involves patterns of action and sensation for the agent, which coincide with the action and perception of the niche. The key point here is that the *agent's field of affordances* (and solicitations) emerges from the dynamics of the MB of the niche, and thereby enables the niche to model sensory causal regularities, or underlying structure of the form of life it constitutes [83], [84].

Now, what are the internal states of the niche? And what are the causal regularities that they model? We suggest that internal states of the niche are a subset of the physical states of the material environment. Namely, the internal states of the niche are the physical states of the environment, which have been modified by the dense histories of different organisms interacting in their shared niche (i.e., histories of active inference). This subset of physical states comes to encode information that is used in the self-evidencing dynamics of organisms. In other words, the ecological niche becomes part of the embodied model parameters that encode the variational or recognition density that the agent uses in active inference. These model parameters encode causal regularities about agents' behaviour, which function as the external, hidden states of the niche, which it models in niche construction.

The internal states of the niche encode information about regularities of the niche-agent(s) relation [40]. This means that the internal states of the niche encode *organism-specific information* (e.g., affordances), not merely any changes to the physical layout. Otherwise, it would mean that the internal states would also encode random changes to the environment (e.g., volcanic eruptions and tsunamis). Hence, the ecological niche is a subset of the physical environment that the organism constructs through reiterated action over time (i.e., active inference). We can see the propensity of the environment to encode organism-specific information as the propensity to change its structure as a function of the actions of the organism (i.e., sensations of the niche) [82]. For instance, a grass patch has a much higher propensity to encode organisms-specific information than a sidewalk made of concrete – hence a grass patch after some time, and recurring actions, might encode a desire path (i.e., will turn into a cultural affordance). The point here is that the niche is always organisms, or species-specific, an Umwelt of sorts [85], whose relational structure consists of affordances, encoded through niche construction (cf. section 3).

The final quantity to define is that of the causal regularities modelled by the niche, and transcribed by its physical layout (the internal states). We have seen that for groups of enculturated agents like humans, internal states of the niche encode affordances that pertain to group behaviour; e.g., what the desire path models is the action possibility to ‘cut corners’. Now, it is a small step from this point to the one that the internal states of the niche track statistical regularities and fluctuations that underwrite the *meaning* of shared intentionality and normative group behaviour (cf. [86], [87], [88]). These regularities are highly abstract, and can only exist – *as causes – for large-scales ensembles like groups of agents*. With the example of the desire path, one can start to appreciate the continuity that obtains between the physics of self-organising systems, the form of pragmatic engagements afforded by constructed niches, and the sort of meanings and intentions that people derive from them.

In summary, the niche transcribes regularities that pertain to *group behaviour* – they are transcribed in the physical layout of the niche – through the active and perceptual dynamics of the agent, which coincides with the perceptual and active dynamics of the niche. These same dynamics entail the landscape of action possibilities (i.e., affordances), which maintains the structural integrity of the agent-niche system – niche dynamics resolve the free energy gradients that are induced by the physical structures of organisms and their niche, and their history of dense interaction and dynamic coupling. In this sense, the robustness of patterns of shared intentionality and enaction of shared meanings is inherited from the robustness of the niche, and *vice versa*.

## 5 Concluding remarks

Variational ecology (VE) is a synthesis of VNE [4], the VANC [40], and the SIF [73], and provides an explanation of collective purposive action and intentionality of living systems – a *physics of sentient systems*. An ecological niche ‘just is’ a structured set of affordances that are shared by agents, which enables its denizens to coordinate purposive action over sometimes vast spatial and temporal distances. The sort of affordances that emerge from niche construction, and that constitute large scale ensembles, carry semiotic and axiological meaning like moral states held in common (cf. [89]) (e.g., this dirt trail ‘means’ cutting through the park, and you shall not be late to your appointment). In effect, there is a deep sense in which affordances are what meanings are [80], and a sense in which meaning is entailed by the existence of groups of agents that share a niche (cf., sense making, [90], [91]). As they engage with the affordances of their niche, ensembles of agents: (i) maintain the structural integrity of the niche through niche construction (and active inference); and (ii) collectively enact a generative model of their relation to the niche, thereby providing an account of the physics of *intentionality*, and especially of *shared intentionality*. VE, then, is also a physics of *interacting minds*.

## Footnotes

2 Note that we use the term ‘ecology’ non-technically, to capture the sorts of interactions among agents and their environment, leading to intentional behaviour, or ‘mind’. We do not appeal directly to the science of ecology, or to human ecology.

3 The destruction of probability gradients simply means that gradient flows will seek out variational free energy minima, where the gradients disappear [77].

4 Bayesian belief updating treats each posterior belief – based upon some data seen so far – as the prior belief for the next set of data. This is formally equivalent to adding the log-evidence (i.e., variational free energy), because adding logs is equivalent to multiplying probabilities and evidence corresponds to the probability of the data, given a model.

5 These abilities are known as *policies* in active inference (please see below).

6 Where Action here denotes a path or time integral of free energy. The Action corresponds to the ‘work done’ in classical mechanics.
